# Corrigendum: Delivery of therapeutic agents by nanoparticles made of grapefruit-derived lipids

**DOI:** 10.1038/ncomms11347

**Published:** 2016-04-20

**Authors:** Qilong Wang, Xiaoying Zhuang, Jingyao Mu, Zhong-Bin Deng, Hong Jiang, Lifeng Zhang, Xiaoyu Xiang, Baomei Wang, Jun Yan, Donald Miller, Huang-Ge Zhang

Nature Communications 4: Article number: 186710.1038/ncomms2886 (2013); Published: 05212013; Updated: 04202016

The financial support for this Article was not fully acknowledged. The Acknowledgements should have included the following:

Huang-Ge Zhang is supported by a Research Career Scientist (RCS) Award.

